# Discordance in left ventricular assessment by CMR vs. echocardiography and potential impact on management of ischaemic left ventricular dysfunction

**DOI:** 10.1093/ehjci/jeaf209

**Published:** 2025-07-17

**Authors:** Tesfamariam Aklilu Betemariam, Holly P Morgan, Stamatis Kapetanakis, Jennifer Mancio, Ebraham Alskaf, Joseph Okafor, Roxy Senior, Matthew Ryan, Amedeo Chiribiri, Divaka Perera

**Affiliations:** British Heart Foundation Centre of Research Excellence at the School of Cardiovascular and Metabolic Medicine & Sciences, King’s College London, Westminster Bridge Road, London SE1 7EH, UK; College of Health Sciences, School of Medicine, Addis Ababa University, 2Q92+P2W, Addis Ababa, Ethiopia; British Heart Foundation Centre of Research Excellence at the School of Cardiovascular and Metabolic Medicine & Sciences, King’s College London, Westminster Bridge Road, London SE1 7EH, UK; St Thomas’ Hospital, Guy’s and St Thomas’ NHS Foundation Trust, Westminster Bridge Road, London SE1 7EH, UK; British Heart Foundation Centre of Research Excellence at the School of Cardiovascular and Metabolic Medicine & Sciences, King’s College London, Westminster Bridge Road, London SE1 7EH, UK; British Heart Foundation Centre of Research Excellence at the School of Cardiovascular and Metabolic Medicine & Sciences, King’s College London, Westminster Bridge Road, London SE1 7EH, UK; Royal Brompton Hospital, Guy’s and St Thomas’ NHS Foundation Trust, Sydney St, London SW3 6NP, UK; Royal Brompton Hospital, Guy’s and St Thomas’ NHS Foundation Trust, Sydney St, London SW3 6NP, UK; British Heart Foundation Centre of Research Excellence at the School of Cardiovascular and Metabolic Medicine & Sciences, King’s College London, Westminster Bridge Road, London SE1 7EH, UK; St Thomas’ Hospital, Guy’s and St Thomas’ NHS Foundation Trust, Westminster Bridge Road, London SE1 7EH, UK; School of Biomedical Engineering and Imaging Sciences, King’s College London, Westminster Bridge Road, London SE1 7EH, UK; British Heart Foundation Centre of Research Excellence at the School of Cardiovascular and Metabolic Medicine & Sciences, King’s College London, Westminster Bridge Road, London SE1 7EH, UK; St Thomas’ Hospital, Guy’s and St Thomas’ NHS Foundation Trust, Westminster Bridge Road, London SE1 7EH, UK

Echocardiography-derived left ventricular ejection fraction (LVEF) provides a quantitative method for grading left ventricular function and is utilized extensively for diagnosis, prognostication, and guiding treatment in ischaemic left ventricular systolic dysfunction (iLVSD). Cardiovascular magnetic resonance imaging (CMR) provides a more reproducible assessment of LVEF but is a limited resource.^1^ There are limited data on the comparability of echocardiography and CMR-derived LVEF in iLVSD, nor is there clear guidance on which modality should be used for clinical decision-making where results are discordant.^2^ This study aimed to (i) evaluate the concordance between these two imaging modalities in LVEF measurement in iLVSD, (ii) compare the prognostic utility of CMR vs. echocardiographic measures of LV volumes and function in this population, and (iii) report the impact of imaging discordance on subsequent therapy, with ICD implantation as a representative example.

Patients with iLVSD (LVEF ≤ 35% and extensive coronary artery disease) enrolled in the REVIVED-BCIS2 trial were eligible if paired pre-randomization transthoracic echocardiography and CMR images were available. Analysis was undertaken by blinded expert readers in two independent core laboratories.^3^ The primary clinical outcome was a composite of all-cause death and aborted sudden death (defined as an appropriate ICD therapy or a resuscitated cardiac arrest). Spearman correlation, Bland–Altman analysis, and coverage probability were performed to assess correlations. A difference of 5% between measurements was pre-defined as acceptable variation. Pitman’s test was used to evaluate whether measurement variance changed as a function of LVEF. Cox proportional hazards models were adjusted for age, sex, diabetes, New York Heart Association class, chronic renal failure, randomized treatment assignment, and coronary jeopardy score. A sensitivity analysis was conducted, restricted to patients undergoing both scans within a 60-day time interval.

Among 373 participants with paired imaging, mean age was 69 ± 9 years, 87% were male, and BMI was 28 ± 5 kg/m². Echocardiography was undertaken an average of 17 days prior to randomization [95% CI −90 to 0 days] and CMR 60 days prior [−133 to −19]. Contrast was used in 17/373 (4.6%) echocardiograms. LVEF was 31.4 ± 9.6% by echocardiography and 25.8 ± 7.9% by CMR (difference −5.5, r = 0.41,*P* < 0.01). Higher indexed volume values were seen with CMR (*Figure 1A*). Only 7.5% of paired LVEF values fell within 5% of each other, with wide limits of agreement (−24.4% to +13.2%) (*Figure 1B*). Greater divergence was observed at lower LVEF values (r = 0.21, *P* < 0.001), regardless of inter-scan interval. The primary endpoint occurred in 137 participants.

**Figure 1:**
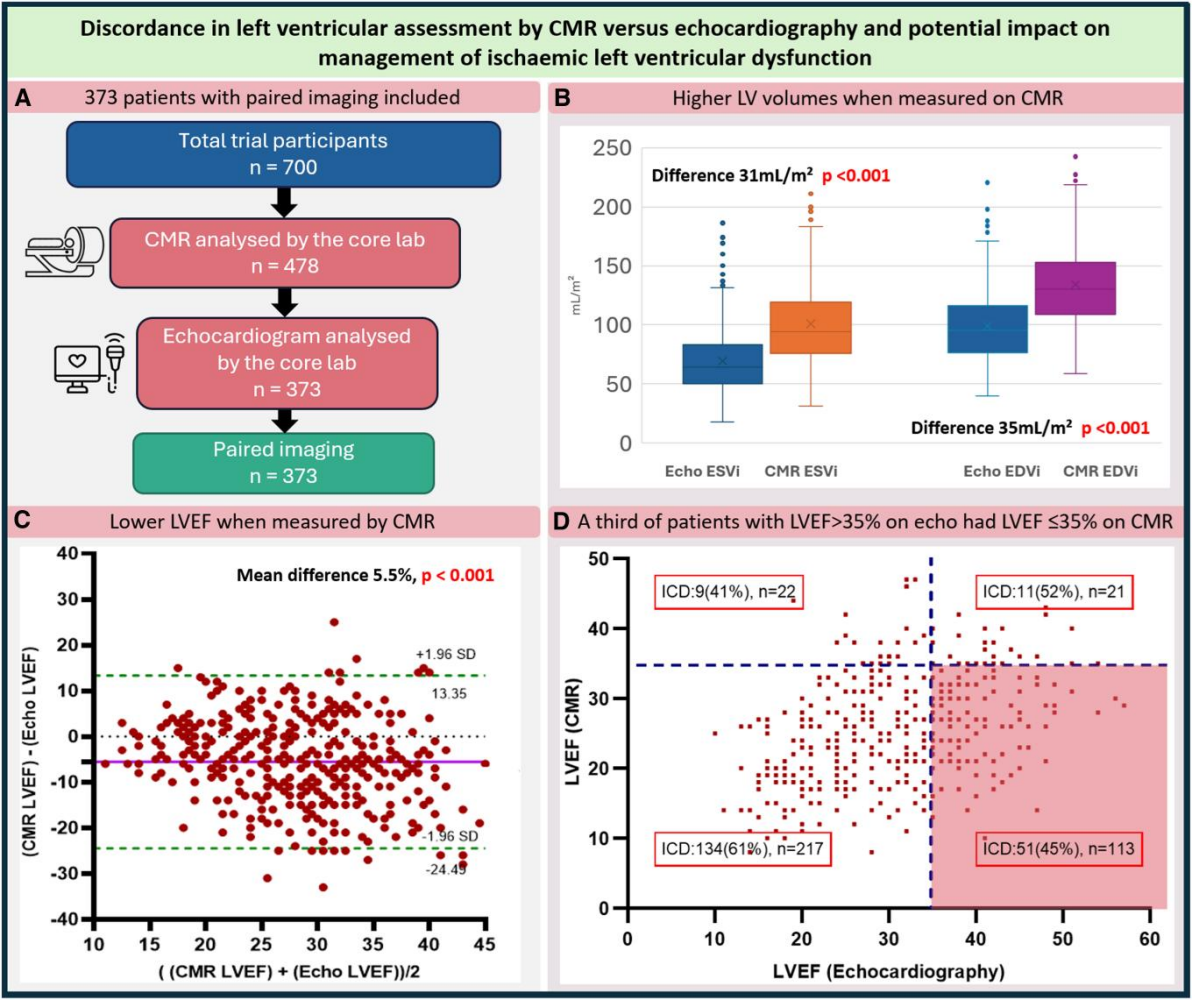
(*A*) Study flow chart. (*B*) Box whisker plots showing echocardiographic and CMR measured ESVi and EDVi. (*C*) Bland–Altman plots comparing CMR and echocardiographic LVEF. *x*-Axis displays the average of the two methods; *y*-axis shows the CMR-echocardiography difference. Dotted lines indicate the 95% limits of agreement. (*D*) Scatter plot of left ventricular ejection fraction (LVEF) measured by echocardiography and CMR. Dashed lines denote the 35% LVEF threshold for each modality. Shading highlights the quadrant in which echocardiography LVEF > 35% but CMR ≤ 35%; Pearson χ² test shows a significant difference between this group and patients with LVEF < 35% by both modalities (χ² = 9.5, *P* < 0.01).

Neither CMR nor echocardiography LVEF was predictive of the primary outcome (adjusted HR 0.935, CI 0.84–1.04, *P* = 0.23 for CMR; and 0.94, 0.85–1.03, *P* = 0.17 for echocardiography) but ESVi predicted outcome with both modalities (HR 1.06, CI 1.01–1.12, *P* = 0.04 for echocardiography; and HR 1.06, CI 1.01–1.11, *P* = 0.04 for CMR). EDVi was only predictive of the primary outcome when measured via CMR.

A third of participants with LVEF < 35% by CMR were classified by echocardiography as having an LVEF ≥ 35% (*Figure 1C*). Among these patients, 45% received an ICD, compared with 61% of patients where both modalities reported the LVEF to be below 35%. Greater discordance was associated with lower ICD implantation rates (*P* < 0.01).

Greater difference between EF measurements was observed in patients with more dilated ventricles, the discordance correlating with both CMR ESVi (β = 0.59, *P* < 0.01), and CMR EDVi (β = −0.48, *P* < 0.01).

In the sensitivity analysis, 199 patients had imaging within 60 days. These results were consistent with the main analysis (EDVi r = 0.72, ESVi r = 0.73, LVEF r = 0.43, all *P* < 0.01).

Our study of patients with severe iLVSD has demonstrated that LVEF measured by CMR is lower than by echocardiography and that the discordance increases with lower LVEF, which is in keeping with previous studies that have found echocardiography to overestimate LVEF compared with CMR.^4,5^ The clinical relevance of this discordance is evidenced by lower rates of ICD implantation in those with LVEF > 35% on echocardiography despite severely impaired function by CMR. Careful consideration needs to be given to interpreting the results of echocardiography and CMR in this context, and future guidelines may clarify how such discordance should be handled.

It is interesting that CMR and echocardiographic ESVi were predictive of the primary outcome, whereas LVEF was not. This may reflect the study inclusion criteria and that once LVEF is severely impaired, volume assessment may aid in further risk stratification.

This study has some limitations. Although most imaging was performed within a short timeframe, the period between tests may have impacted measurement variation. As recruitment was limited to patients with LVEF ≤ 35%, the results cannot be extrapolated to those with better LVEF. Finally, as this was a retrospective observational study, we do not know what led to the difference in implant rates in discordant vs. concordant groups.

In conclusion, CMR and echocardiography LV measurements were only modestly correlated in a population with ischaemic left ventricular systolic dysfunction. This discordance should be considered when assessing patients for therapeutic interventions.

## Data Availability

The data supporting the findings of this study will be made available on reasonable request to the corresponding author, via email.
